# How do various strategies for returning residues change microbiota modulation: potential implications for soil health

**DOI:** 10.3389/fmicb.2024.1495682

**Published:** 2025-01-21

**Authors:** Nan Jiang, Zhenhua Chen, Yi Ren, Shichang Xie, Zimeng Yao, Dongqi Jiang, Yulan Zhang, Lijun Chen

**Affiliations:** ^1^CAS Key Laboratory of Forest Ecology and Silviculture, Institute of Applied Ecology, Chinese Academy of Sciences, Shenyang, China; ^2^Shenyang National Field Scientific Observation and Research Station of Farmland Ecosystem, Shenyang, China; ^3^Iotabiome Biotechnology Inc., Suzhou, China; ^4^Suzhou Medical College, Soochow University, Suzhou, China; ^5^University of Chinese Academy of Sciences, Beijing, China

**Keywords:** residue returning, soil health, soil microbiomes, nutrient cycling, antibiotic resistance genes, pathogen

## Abstract

**Introduction:**

Residue incorporation is a crucial aspect of anthropogenic land management practices in agricultural fields. However, the effects of various returning strategies on the soil microbiota, which play an essential vital role in maintaining soil health, remains largely unexplored.

**Methods:**

In a study conducted, different residue management strategies were implemented, involving the application of chemical fertilizers and residues that had undergone chopping (SD), composting (SC), and pyrolysis (BC) processes, with conventional fertilization serving as the control (CK).

**Results and discussion:**

Using metagenomic sequencing, the analysis revealed that while all residue returning strategies had minimal effects on the diversity (both *α* and *β*) of microbiota, they did significantly alter microbial functional genes related to carbon (C), nitrogen (N), phosphorus (P), and sulfur (S) cycling, as well as the presence of antibiotic resistance genes (ARGs) and pathogens. Specifically, chopped residues were found to enhance microbial genes associated with C, N, P, and S cycling, while composted residues primarily stimulated C and S cycling. Furthermore, all residue treatments resulted in a disruption of relationships among nutrient cycles, with varying degrees of impact observed across the different management strategies, with the sequence of impact being SD < SC < BC. Moreover, the residue additions resulted in the accumulation of ARGs, while only SC caused an increase in certain pathogens. Finally, through analyzing the correlation network among indices that exhibited active responses to residue additions, potential indicators for functional changes in response to residue additions were identified. This study further offered recommendations for future cropland management practices aimed at enhancing soil health through microbiomes.

## Introduction

Soil health has been broadly defined as the ongoing capacity of a living soil to support and sustain plant, animals and humans (Doran, 2002; Tahat et al., 2020). The presence of microbiota is necessary for maintaining soil health. The soil microbiome plays a crucial role in enhancing crop yield, safeguarding and nourishing plants, and providing essential ecosystem services such as climate regulation, water purification, and erosion prevention (Choi et al., 2017; Bahram et al., 2018). Moreover, the soil microbiome plays an essential role in driving soil processes, including the cycling of carbon (C), nitrogen (N), phosphorus (P), sulfur (S), and other nutrients, and further determines fertility evolution (Fierer, 2017; Wang et al., 2024). Indigenous microbiomes have been shown to be effective in combating soil-borne plant pathogens, thereby facilitating their establishment or persistence (Schlatter et al., 2017; Banerjee and van der Heijden, 2022). An imbalance in the composition and function of microbial communities results in a decline in soil health (Tahat et al., 2020). Regrettably, an ideal soil microbiome for maintaining healthy soils may not exist, just like the high variation observed in the gut microbial community of healthy humans (Falony et al., 2016; Fierer et al., 2020). Therefore, understanding the microbial taxa and their functional characteristics is crucial for monitoring changes in soil health in response to shifts in soil conditions (Fierer et al., 2020).

The global population is estimated to reach 8.9 billion by the year 2050, resulting in high demands for agricultural products (Lichtfouse et al., 2009). The expansion of intensive agriculture globally to address food demands has led to soil degradation and environmental challenges in certain agroecosystems which has negatively impacted the soil microbiome and overall soil health (Bahram et al., 2018; Zhu and Penuelas, 2020). For example, long-term fertilization regimes excluding the return of organic materials have led to notable alterations in the microbiome composition, hastened nutrient cycling processes like C and N biotransformation, and resulted in further C and N loss (Hallin et al., 2009; Zhu and Penuelas, 2020; Yang et al., 2022). Alternatively, the prolonged use of chemical fertilizers has been found to diminish the diversity and functionality of P-cycling microbial communities, consequently decreasing their capacity to supply P for plant uptake (Chen et al., 2017; Chen et al., 2019). Hence, the significance of crop residue management as a key component of sustainable agriculture is progressively gaining global attention due to its dual benefits of enhancing soil health and increasing crop yield (Turmel et al., 2015; Tahat et al., 2020). These outcomes are contingent upon the diversity of microbial communities and their associated functional genes (Guerra et al., 2021).

Undoubtedly, various strategies for returning residues have diverse effects on soil microorganisms. Both the original and composted residue offer distinct complex substrates for soil microorganisms to mineralize, leading to the eventual availability of nutrients (Hartmann and Six, 2023). The extended application of crop residues, whether integrated into the soil or maintained on the soil surface, significantly enhances the microbial processes involved in C sequestration (Govaerts et al., 2009; Turmel et al., 2015), N provision (Wu et al., 2021a; Yuan et al., 2022), and P transformation (Chen et al., 2017; Wu et al., 2021b) in comparison to conventional fertilization practices. The composted residue undergoes a microbe-mediated, thermophilic, and aerobic fermentation process, leading to partial humification similar to the natural decomposition of recalcitrant components in soils (Sánchez et al., 2017). In this case, the composted residues provide more stable organic matter and host a diverse microbial community in contrast to the original residues, consequently establishing a distinct soil function (Sánchez et al., 2017; Hartmann and Six, 2023). The soil microbial community exhibited immediate changes following the addition of composted residue, with a notable increase observed in species involved in the degradation of organic matter rather than in P-turnover bacteria (Kraut-Cohen et al., 2023; Pu et al., 2023). Composted residues have been observed to have a reduced impact on microbial communities compared to original residues in previous studies (Maltais-Landry et al., 2015; Pu et al., 2023). Additionally, they have shown the potential to suppress soil-borne diseases (Tilston et al., 2002). Unlike the aforementioned residue returning strategies, the utilization of crop residue post-pyrolysis (i.e., biochar) results in a rich recalcitrant C content, with up to 97% of the biochar C being recalcitrant, and thereby enhancing C sequestration and facilitating nutrient retention through ion adsorption (Wang et al., 2016; Hartmann and Six, 2023). As a result, biochar is frequently used to improve soil health, but its effects on the soil microbiome can be inconsistent or contradictory due to the diverse soil types and properties of biochar in different studies (Li et al., 2020; Hartmann and Six, 2023). Some studies have indicated that the application of biochar can influence soil properties and subsequently impact soil microbial communities (Jindo et al., 2012; Song et al., 2019). Conversely, other research has shown a reduction in fungal diversity in response to high pH caused by biochar application (Kamble et al., 2014; Li et al., 2020). Recent studies on the effects of crop residue returning have predominantly concentrated on microbial diversity or specific aspects of microorganisms, often neglecting the functional roles of these microbes. Consequently, further investigation on the microbiome is required to scrutinize the varying effects of crop residue return strategies on soil health.

Here, we present a metagenomics analysis of the soil microbiome under conventional mineral fertilizers and three residue returning strategies, including chopped maize straw, its derived compost, and biochar. Different strategies for returning residues contain different C sources, nutrients or microbial species, thus we hypothesized that returning residues can enhance soil health by increasing the microbial potential to: (1) recycle nutrients and energy with all residue returns; (2) decompose organic matter in both original and composted residue treatments; (3) suppress pathogens following the returning of composted residues; and (4) promote the mineralization of soil organic matter with the addition of biochar. Furthermore, as a result of the extensive shotgun sequencing, a substantial volume of novel data is produced, revealing the potential microbial genes and pathways present in soils that could play a role in soil health.

## Materials and methods

### Site description and soil sampling

The field trial was conducted at the National Field Observation and Research Station of Shenyang Agro-ecosystems in the Liaohe Plain of northeast China (N41°31′, E123°24′). The region experiences a temperate, humid, continental monsoon climate characterized by an average temperature ranging from 7.0 to 8.1°C, a frost-free period lasting between 153 and 180 days, and annual precipitation levels between 575 and 684 mm. The soil is categorized as meadow brown soil according to Soil Taxonomy, with a soil pH of 5.72, 0.45 g of P kg^−1^ dry soil, and 1.10 g N kg^−1^ dry soil.

Prior to 2010, the region was subjected to conventional tillage practices with uninterrupted maize cultivation for over three decades, during which the aboveground portions of maize straws were eliminated post-harvest. From 2010 to 2014, maize cultivation persisted without the application of fertilizers to achieve optimal uniformity in soil fertility. Four treatments, each with four replicates (4.95 m × 30 m), were organized following a complete randomized block design in April 2015. The treatments included: (1) conventional tillage (CK), involving the removal of maize straws; (2) the application of biochar derived from maize straws on-site (BC); (3) the application of composted maize straws (SC); (4) the direct addition of chopped maize straws on-site (SD). The quantity of maize straws remained consistent across all treatments, approximately totaling 6,000 kg ha^−1^ y^−1^. The efficiency of charring and decomposition was estimated at around 30% based on dry weight measurements, with the application rate of biochar or compost being approximately 2000 kg ha^−1^ y^−1^. The properties of additives were previously documented (Supplementary Table S1) (Pu et al., 2023). All treatments involved the application of NPK fertilizers (urea: 180 kg N ha^−1^; P_2_O_5_: 75 kg ha^−1^; K_2_O: 75 kg ha^−1^), following by tilling the fields to incorporate the additives into the soil (Pu et al., 2023).

Soil samples (0–10 cm depth) were obtained post-harvest in October using a 5 cm diameter auger. For each plot, five soil cores were randomly collected and combined into a composite sample, which was promptly homogenized using a 2-mm sieve. A fraction of each soil sample was stored at −80°C for DNA extraction, and another fraction was subjected to air drying for further analysis.

### DNA extraction and metagenomic sequencing

Sixteen soil samples were subjected to metagenomic sequencing. For each sample, genomic DNA was extracted from 1.0 g of fresh soil using the FastDNA® Spin Kit for soil (Qbiogene, CA, United States) following the manufacturer’s protocol. The concentration and purity of DNA were determined using the TBS-380 fluorometer and Nanodrop2000 spectrophotometers, respectively. Paired-end sequencing was conducted using an Illumina Hiseq Xten platform (Illumina Inc., San Diego, CA, USA) at Majorbio Bio-Pharm Technology Co., Ltd. (Shanghai, China) with the HiSeq X Reagent Kit following the guidelines provided by the manufacturer.^1^

### Metagenomic assemble and functional annotation

Adaptor sequences were removed from both the 3′- and 5′-ends of Illumina reads using SeqPrep.^2^ Sequences with a length shorter than 50 bp, a quality value below 20, or containing N bases were removed using Sickle.^3^ The clean sequences were then assembled using MEGAHIT (Li et al., 2015b), and contigs that were more than 300 bp were retained for further analyses. Open reading frames (ORFs) were predicted using MetaGene (Noguchi et al., 2006), and were further clustered at 95% identity level with 90% coverage using CD-HIT (Fu et al., 2012). The longest ORF in each cluster was extracted to compose the non-redundant (nr) gene catalog, and the number of reads mapping to genes was calculated in each sample using SOAP2 (Li et al., 2009). All nr genes were searched against the National Center for Biotechnology Information (NCBI) database to reveal the taxonomic information using BLASTP (Version 2.2.28+) with an *e*-value cutoff of 1e^−5^(Altschul et al., 1997). Kyoto Encyclopedia of Genes and Genomes (KEGG) annotations were also performed using BLASTP against the KEGG database^4^ (Xie et al., 2011) with an *e*-value cutoff of 1e^−5^. All analyses were performed on marker abundances normalized to reads per kilobase per million reads (RPKM).

### Taxonomic annotation

We use clean sequences for taxonomic annotation. First, the each of the clean sequences was aligned against UniRef100 using DIAMOND (Buchfink et al., 2015) with parameter e-value 1e^−5^, and the top 10% of alignment results are selected for downstream analysis. Second, from species to phylum, a taxon is selected for each sequence if more than 50% alignment results support it. Finally, the abundance of a taxon is the sum of all sequences supporting it and is normalized by the total sequences for each sample.

The potential pathogens for both plants and animals are selected from the taxonomic annotation if a taxon is included in the pathogen-host interactions database including 264 pathogens (Winnenburg et al., 2006).

### Annotation of CNPS functional genes

The NPS functional genes were downloaded from NCycDB, PCycDB and SCycDB, respectively (Tu et al., 2019; Yu et al., 2021; Zeng et al., 2022). Then the sequences of NPS functional genes were merged using CD-HIT (Fu et al., 2012) with a 90% sequence identity threshold to create the non-redundant database. Clean sequences are aligned against the non-redundant database using DIAMOND with parameter e-value 1e^−5^. The carbohydrate active enzyme (CAZyme) was annotated using DIAMOD against CAZyDB provided by dbCAN2 with default parameters (Huang et al., 2017). The abundance values of the CAZymes were assigned by counting the clean sequences from each sample which hit them. The KEGG Orthology (KO) information of carbon cycling functional genes is further extracted based on the KEGG pathways related to carbon metabolism. Clean sequences are aligned against the UniRef100 using DIAMOND and the annotation of the C functional genes are obtained if the hits of UniRef100 have KO number related the carbon metabolism pathways of KEGG.

### Antibiotic resistance genes analysis

The annotation and abundance of antibiotic resistance genes are made using the ARGs-OAP pipeline v3.2.4 (Yin et al., 2022). Specifically, the clean sequences were initially searched for ARGs against SARG database version 3.0 by BLAST + (version 2.12.0), employing the following parameters: similarity ≥ 80%, e-value ≤ 1e ^− 7^ and query coverage ≥ 75%. Subsequently, the abundance of ARGs was determined by the ratio of ARG copies to 16S rRNA copies, utilizing the ARGs-OAP method (Li et al., 2015a).

### Statistical analysis of sequencing data

The *Kruskal Wallis* test (*p* < 0.05) was employed to examine differences in the abundance of phyla, genera, KO, and enzymes utilizing the agricolae package in R.^5^ The Bray–Curtis similarity of both taxonomic composition and KO composition was assessed through Principal Component Analysis (PCA) using the vegan package in R. Spearman correlations were calculated using the Hmisc package in R. The correlation networks were further established when the |Spearman correlation coefficient (r)| > 0.6 and a *p* value <0.05. The networks were modularized and visualized using the Cytoscape (version 3.10.2) (Bader and Hogue, 2003; Shannon et al., 2003; Assenov et al., 2008).

## Results

### Taxonomic distribution of the metagenomes

Shotgun metagenomic sequencing yielded a total of 238.24 Gb clean data, averaging 14.89 Gb per sample. The assembly of contigs for each sample varied between 598,234 and 882,061, resulting in the prediction of a total of 14.35 million ORFs. The details of sequencing are outlined in Supplementary Table S2.

PERMANOVA analysis indicated that there were no significant differences in microbial profiles across the various treatments (*R*^2^ = 0.19, *p* = 0.50). The predominant domain was Bacteria (95.2–95.9%), followed by Archaea (0.57–0.92%), Eukaryota (0.37–0.84%) and Viruses (0.02–0.12%). A significant decrease in the relative abundance of Archaea was observed in treatments with residue inputs compared to CK (One-way ANOVA with Duncan test, *p* < 0.05).

Among the 163 phyla identified, Proteobacteria (33.4% ± 1.2%), Actinobacteria (17.3% ± 2.64%), Acidobacteria (15.5% ± 1.37%), and Chloroflexi (6.88% ± 0.69%) were found to be dominant (the relative abundance >5%), followed by Gemmatimonadetes (4.71% ± 0.42%), Bacteroidetes (3.40% ± 0.57%), Planctomycetes (3.26% ± 0.32%), Verrucomicrobia (2.81% ± 0.29%), Candidatus Rokubacteria (1.93% ± 0.57%), Cyanobacteria (0.97% ± 0.14%), Firmicutes (0.86% ± 0.04%), Nitrospirae (0.82% ± 0.10%), and Thaumarchaeota (0.50% ± 0.10%).

**Figure 1:**
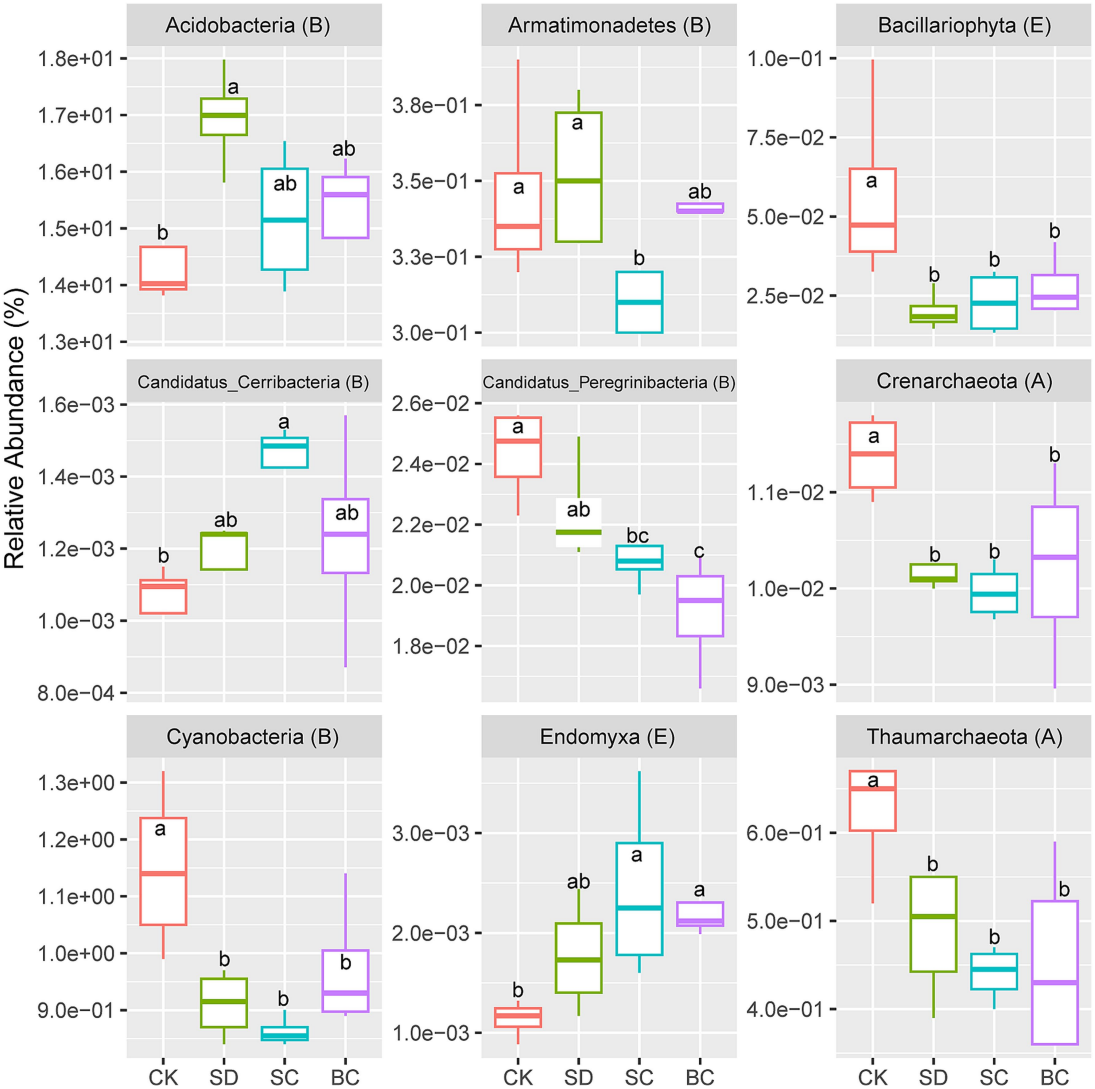
The specific phyla that exhibited significant differences across different treatments. Different lowercase letters indicate the significant differences between treatments as identified by one-way ANOVA with Duncan’s test (*p* < 0.05). CK, without addition of organic matter; BC, the addition of biochar converted from maize straw; SC, the addition of composted maize straw; SD: the addition of chopped maize straw. The letters “A,” “B,” and “E” in brackets indicate the archaeal, bacterial and eukaryotic domains, respectively.

The results of the one-way ANOVA with *Duncan*’s test suggested a significant decrease in the relative abundance of two archaeal phyla Crenarchaeota and Thaumarchaeota, one eukaryotic phylum Bacillariophyta, and two bacterial phyla Cyanobacteria and Candidatus_Peregrinibacteria significantly decreased under all residue treatments when compared to the CK treatment. Conversely, the relative abundance of the eukaryotic phylum Endomyxa exhibited an opposite trend (Figure 1). In addition, there was a notable increase in the relative abundance of two bacterial phyla Acidobacteria and Candidatus_Cerribacteria in SD and SC, respectively. Conversely, the relative abundance of the bacterial phylum Armatimonadetes was significantly decreased in the SC (Figure 1).

### Functional distribution of the metagenomes

**Figure 2:**
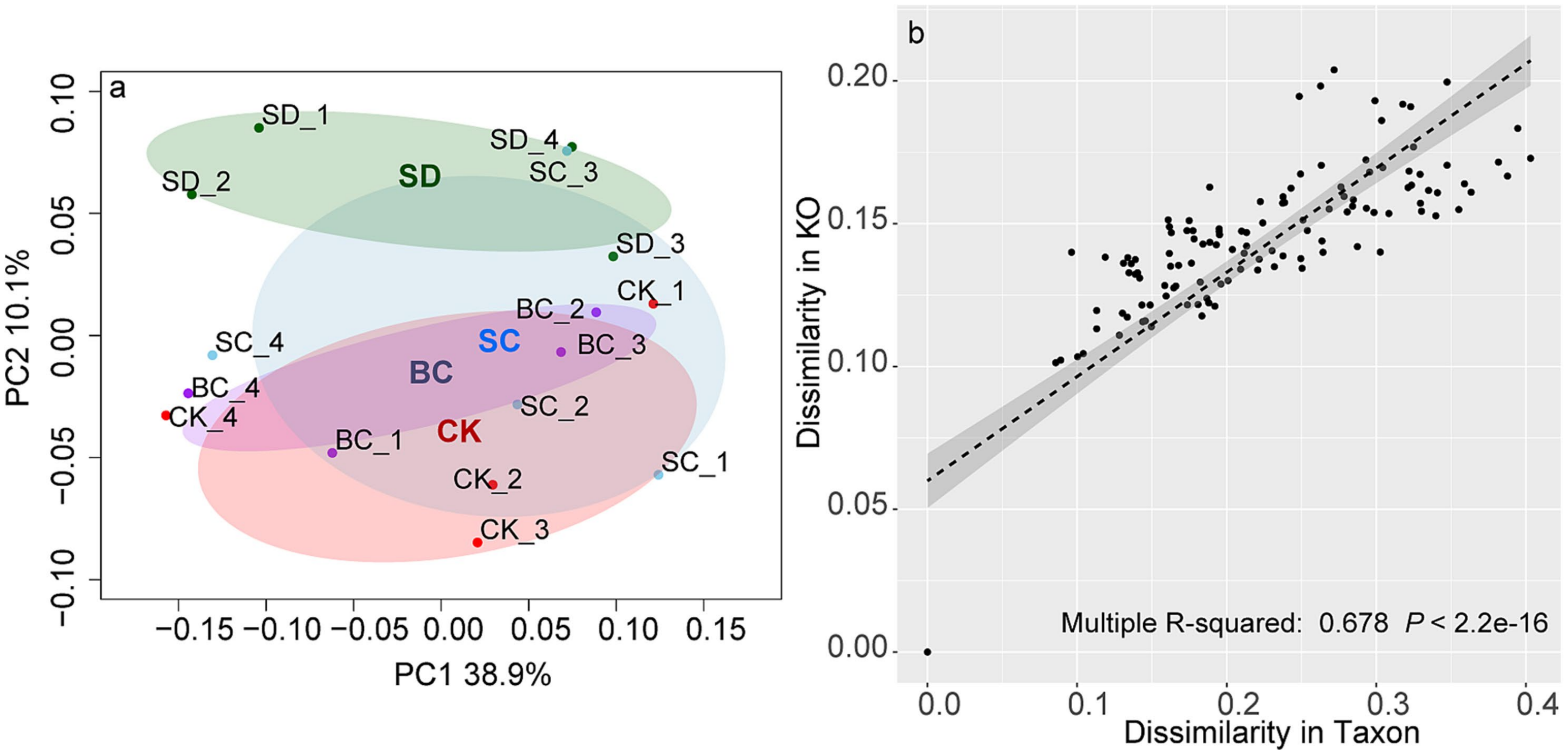
The functional profiles **(A)** across the treatments and their correlations with microbial profiles as determined by Bray-Curtis distance matrices **(B)**, The result of pairwise comparison CK, without addition of organic matter; BC, the addition of biochar converted from maize straw; SC, the addition of composted maize straw; SD: the addition of chopped maize straw. The pairwise ANOSIM R statistics calculated between treatments (Permutations = 999) are as follows: All: *R*^2^ = 0.678 *p* = 0.079, CK-BC: *R*^2^ = 0.025 *p* = 0.918, CK-SC: *R*^2^ = 0.054 *p* = 0.523, CK-SD: *R*^2^ = 0.518 *p* = 0.057, BC-SC: *R*^2^ = 0.051 *p* = 0.848, BC-SD: *R*^2^ = 0.599 *p* = 0.030, SC-SD: *R*^2^ = 0.421 *p* = 0.087. *R*^2^ indicates dissimilarities among the treatments.

Pairwise comparisons showed that the significant differences in functional profiles were primarily driven by SD (Figure 2A), though there was a significant association between functional profiles and microbial profiles (Figure 2B).

### Functional genes involved in the cycling of C, N, P, S

**Figure 3:**
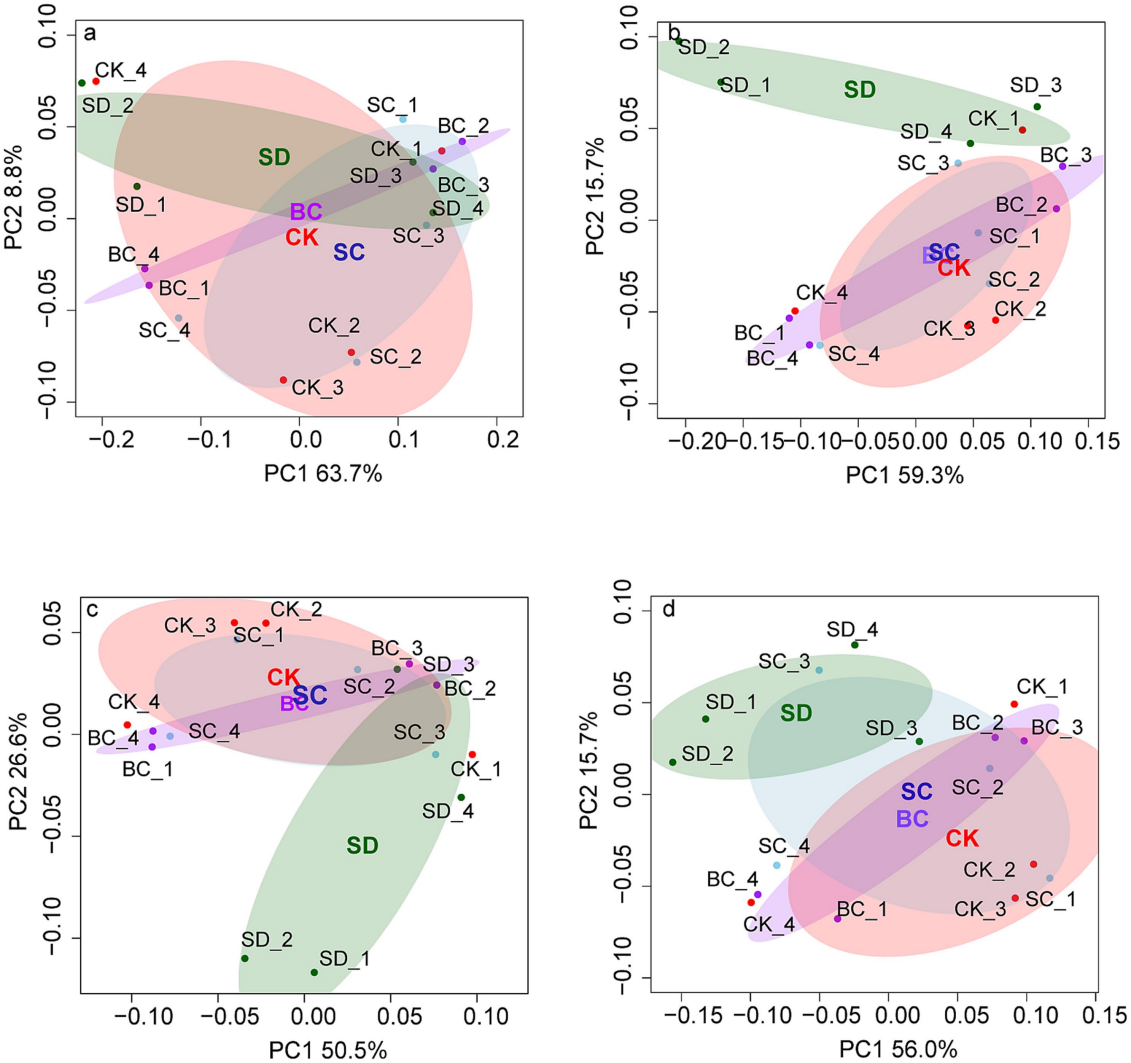
The functional profiles of carbon **(A)**, nitrogen **(B)**, phosphorus **(C)**, and sulfur **(D)** cycling across the treatments based on Bray-Curtis distance matrices. CK, without addition of organic matter; BC, the addition of biochar converted from maize straw; SC, the addition of composted maize straw; SD: the addition of chopped maize straw. For each group, an ellipse was constructed with a confidence level of 0.95. The pairwise ANOSIM R statistics calculated between treatments (Permutations = 999) are shown as follows: **(A)** All: *R*^2^ = 0.318 *p* = 0.172, CK-BC: *R*^2^ = 0.043 *p* = 0.824, CK-SC: *R*^2^ = 0.053 *p* = 0.657, CK-SD: *R*^2^ = 0.464 *p* = 0.088, BC-SC: *R*^2^ = 0.025 *p* = 0.942, BC-SD: *R*^2^ = 0.399 *p* = 0.089, SC-SD: *R*^2^ = 0.286 *p* = 0.148; **(B)** All: *R*^2^ = 0.317 *p* = 0.180, CK-BC: *R*^2^ = 0.026 *p* = 0.827, CK-SC: *R*^2^ = 0.028 *p* = 0.766, CK-SD: *R*^2^ = 0.425 *p* = 0.092, BC-SC: *R*^2^ = 0.013 *p* = 1.000, BC-SD: *R*^2^ = 0.448 *p* = 0.081, SC-SD: *R*^2^ = 0.345 *p* = 0.109; **(C)** All: *R*^2^ = 0.441 *p* = 0.044, CK-BC: *R*^2^ = 0.025 *p* = 0.918, CK-SC: *R*^2^ = 0.054 *p* = 0.523, CK-SD: *R*^2^ = 0.518 *p* = 0.057, BC-SC: *R*^2^ = 0.051 *p* = 0.848, BC-SD: *R*^2^ = 0.599 *p* = 0.030, SC-SD: *R*^2^ = 0.421 *p* = 0.087; **(D)** All: *R*^2^ = 0.146 *p* = 0.673, CK-BC: *R*^2^ = 0.020 *p* = 1.000, CK-SC: *R*^2^ = 0.054 *p* = 0.768, CK-SD: *R*^2^ = 0.179 *p* = 0.291, BC-SC: *R*^2^ = 0.040 *p* = 0.944, BC-SD: *R*^2^ = 0.156 *p* = 0.372, SC-SD: *R*^2^ = 0.147 *p* = 0.376.

Likewise, significant differences in genetic profiles of N, P, and S cycling were also predominantly influenced by SD, whereas genetic profiles of C cycling exhibited similarity across all treatments (Figure 3).

**Figure 4:**
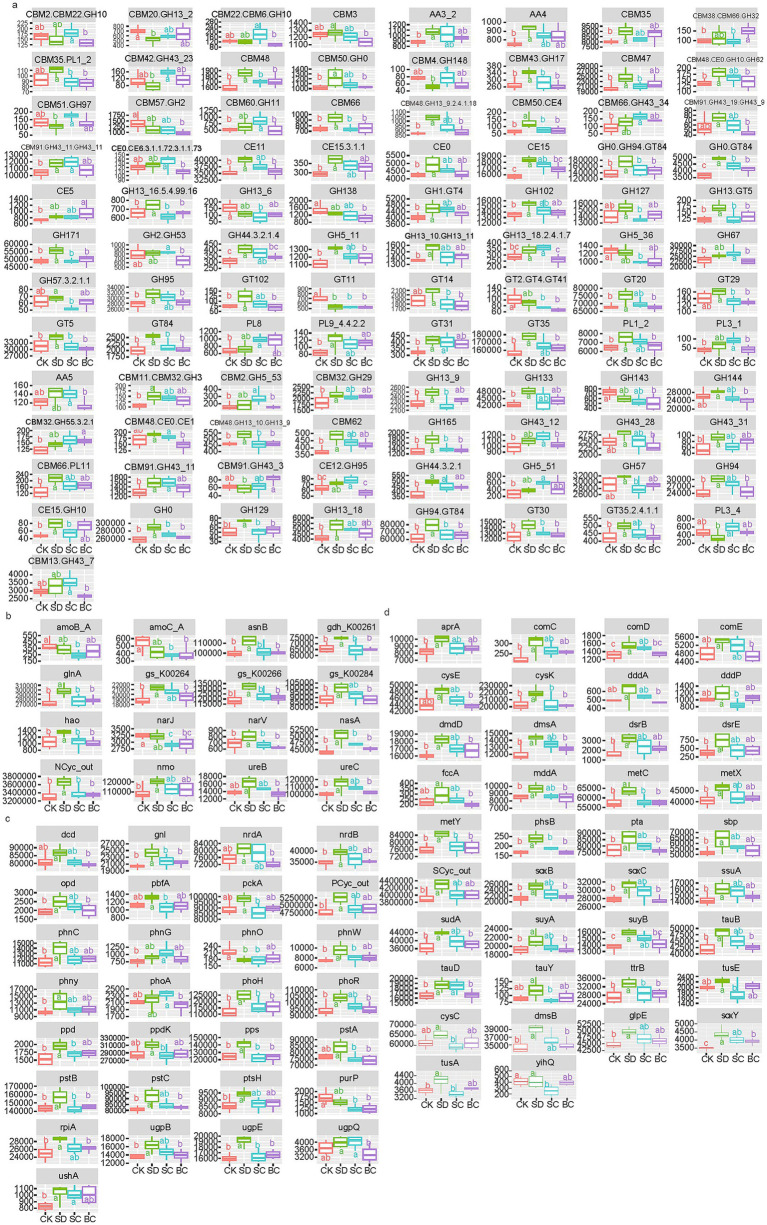
Gene families exhibiting notable differences in reads between treatments for carbon **(A)**, nitrogen **(B)**, phosphorus **(C)**, and sulfur **(D)** cycles. CK: without addition of organic matter; BC: the addition of biochar converted from maize straw; SC: the addition of composted maize straw; SD: the addition of chopped maize straw. The lowercases indicate the significant difference between treatments one-way ANOVA with Duncan’s test (*p* < 0.05). GH, Glycoside hydrolases; CBM, Carbohydrate-binding modules; CE, Carbohydrate esterases; AA, Auxiliary activities; PL, Polysaccharide lyases; GT, Glycosyl transferases.

A total of 97 subfamilies of C cycling exhibited significant differences between treatments (Figure 4A), with over 72% of them being classified under Glycoside Hydrolases (GHs). Within these GHs, 42.9% were found to contain Carbohydrate-Binding Modules (CBMs), while 17.1% were found to contain Carbohydrate Esterases (CEs).

A total of 16 genes of N cycling exhibited significant differences among the treatments (Figure 4B). More than 56% of these processes pertain to organic degradation and synthesis, encompassing genes such as ureC, ureB, nmo, asnB, glnA, etc., followed by nitrification (amoC, amoB, and hao), denitrification (narJ), and assimilatory nitrate reduction (nasA).

A total of 29 gene families of P cycling exhibited significant differences between the treatments (Figure 4C). Approximately 39.3% of these genes involving in crucial microbial processes related to the regulation, transportation, and absorption of P sources from the environment, including organic phosphoester hydrolysis, P transporters, and two-component systems. The other processes were accountable for cellular P metabolic pathways involved in the synthesis of organic P compounds, including the pentose phosphate pathway, phosphonate and phosphinate metabolism, phosphotransferase system, purine metabolism, pyrimidine metabolism, and pyruvate metabolism.

Significant differences were detected in 38 genes related to S cycling among the treatments (Figure 4D). Among these genes, there were 12 organic sulfur transformation genes and eight linkages between inorganic and organic S transformation genes, followed by S oxidases system, S oxidation, and S reduction, etc.

**Figure 5:**
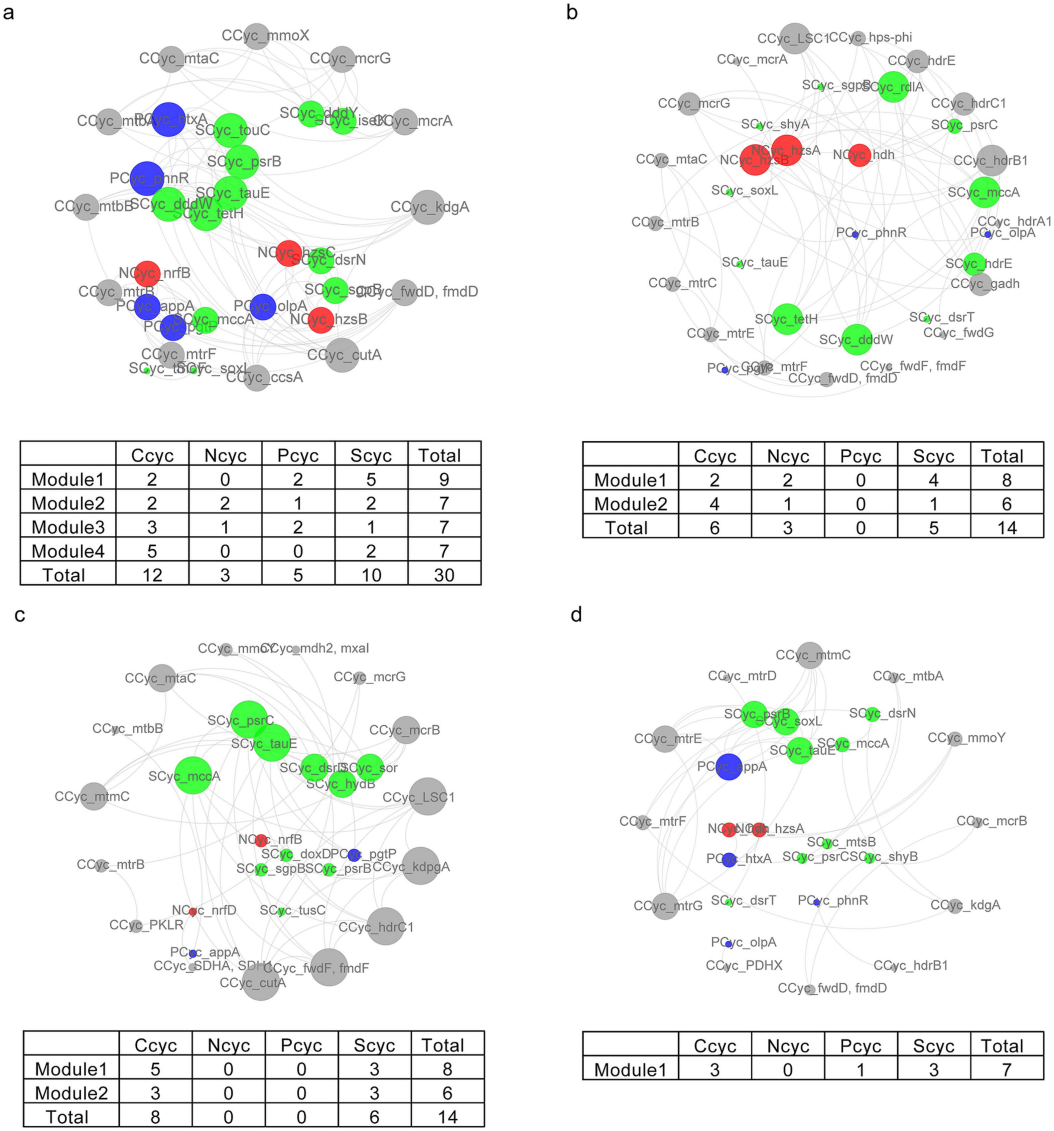
The correlation network of caron (gray), nitrogen (red), phosphorus (blue), and sulfur (green) cycling in CK **(A)**, SD **(B)**, SC **(C)**, and BC **(D)**. The edges indicate significant relationships between two nodes. The sizes of nodes and names are ordered according to the clustering coefficient. The tables display the gene counts related to nutrient cycling within each module. CK, without addition of organic matter; BC, the addition of biochar converted from maize straw; SC, the addition of composted maize straw; SD, the addition of chopped maize straw.

Additionally, the application of all residue additions led to significant alterations in the relationships between C, N, P, and S cycling, characterized by a decrease in clustering coefficient, average number of neighbors, and network density (Supplementary Table S3). The CK, BC, SC, and SD treatments contained four, one, two, and two modules, respectively, representing 94, 26, 48, and 41% of nodes in each module (Figure 5). In contrast to CK, which harbored significant correlations between C, N, P, and S cycling, the predominant distribution included C, N, and S cycling in SD, C and S cycling in SC, and C, P, and S cycling in BC (Figure 5).

### Antibiotic resistance genes

**Figure 6:**
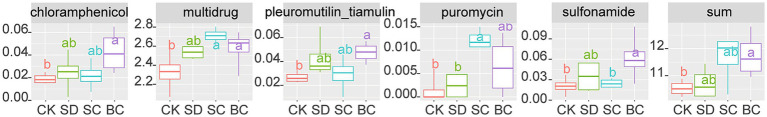
Antibiotic resistance genes (defined as the ratio of antibiotic resistance gene copies to 16S rRNA copies) that exhibit significant differences among the treatments. CK, without addition of organic matter; BC, the addition of biochar converted from maize straw; SC, the addition of composted maize straw; SD, the addition of chopped maize straw. Sum: the total of antibiotic resistance genes. The lowercases indicate the significant difference between treatments one-way ANOVA with Duncan’s test (*p* < 0.05).

A total of 26 antibiotic resistance genes were identified among the samples. When comparing BC to CK, significant increases in chloramphenicol, multidrug, pleuromutilin_tiamulin, and sulfonamide were observed. Conversely, significant increases in multidrug and puromycin were noted in SC (Figure 6).

### Potential pathogens

**Figure 7:**
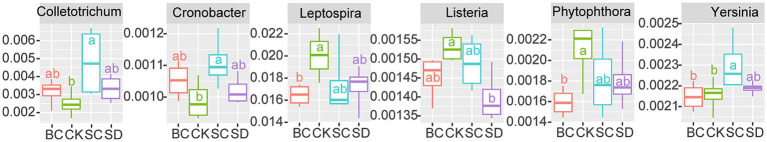
Pathogens (defined as the ratio of pathogen copies to 16S rRNA copies) that exhibit significant differences among the treatments. CK: without addition of organic matter; BC, the addition of biochar converted from maize straw; SC, the addition of composted maize straw; SD, the addition of chopped maize straw. The lowercases indicate the significant difference between treatments one-way ANOVA with Duncan’s test (*p* < 0.05).

A total of 58 pathogens were identified across the samples. When compared to CK, significant increases in Colletotrichum, Cronobacter, and Yersinia were detected in SC, significant decreases in Leptospira and Phytophthora were observed in BC, and a significant decrease in Listeria was found in SD (Figure 7).

### Correlations between microbiomes with changes in response to residue additions

**Figure 8:**
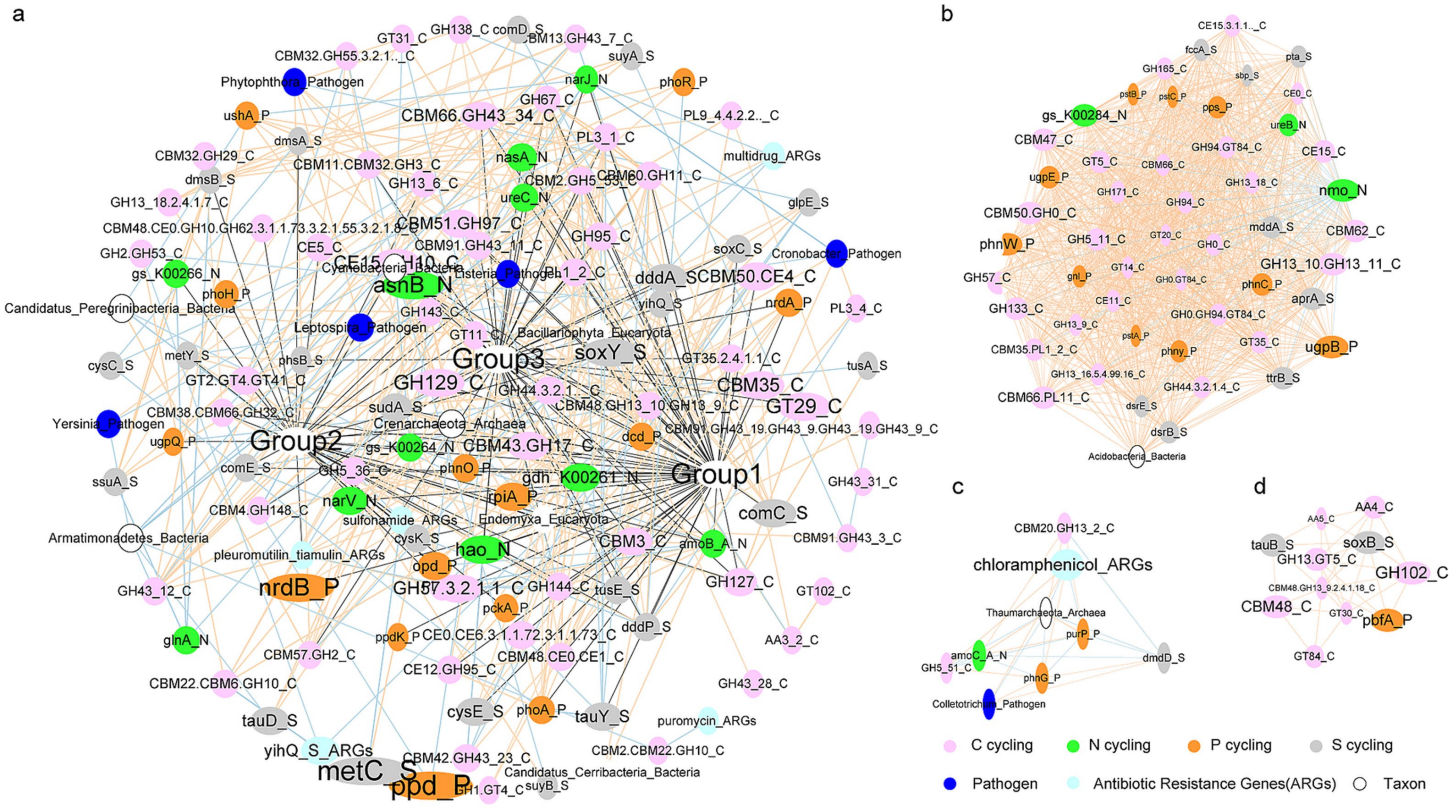
The correlation network among the indices that were changed in response to residue additions. The edges indicate significant relationships between two nodes. The sizes of nodes and names are ordered according to clustering coefficient. **(A)** Correlation network among all the changed indices. Modules labeled Group1-Group3 in **(A)** are depicted in **(B,D)**, respectively.

Significant and strong correlations were observed among the indices that were altered in response to residue additions (Supplementary Figure S1). The correlation network comprised 195 nodes and 2,461 edges. Within this network, two genes associated with P cycling (nrdB and ppd), one gene associated with N cycling (asnB), one gene associated with S cycling (metC), and one gene associated with C cycling (GH102) were identified as playing important roles (Figure 8). Three modules were identified, consisting of 51 nodes and 1,090 edges (Figure 8B, group1), 9 nodes and 26 edges (Figure 8C, group2), and 11 nodes and 30 edges (Figure 8D, group3). In addition to C, N, P, and S cycling genes, Group1 included the bacterial phylum Acidobacteria besides C, N, P, and S cycling, Group2 included archaeal phylum Thaumarchaeota, pathogen Colletotrichum, and antibiotics resistance gene chloramphenicol (Figure 8).

## Discussion

### The impact of residue additions on functional profiles outweighed that on microbial communities

The study investigated the effects of residue application over a five-year period on the diversity of microbial communities in soil. While overall *α* and *β* diversity remained largely unaffected, certain populations, including Archaea, Cyanobacteria, Peregrinibacteria, and Bacillariophyta, experienced a significant reduction in abundance when exposed to added residue. These microorganisms are vital for the carbon cycle in soil, especially in challenging or oligotrophic environments. For instance, Cyanobacteria are crucial for oxygenic photosynthesis and carbon fixation (Knoll, 2008; Sánchez-Baracaldo et al., 2022). Likewise, archaeal, peregrinibacterial, and bacillariophytal populations also possess highly efficient pathways for carbon fixation (Wrighton et al., 2016; Shnyukova and Zolotariova, 2017; Baker et al., 2020). Conversely, the relative abundance of Endomyxa increased with residue addition, aligning with prior research indicating its responsiveness to organic inputs (Zhao et al., 2019; Fiore-Donno et al., 2020). In summary, microorganisms thriving in low-nutrient environments exhibited a significant decline in abundance following residue additions, while the opposite trend was observed for other microbial populations.

In contrast to the microbial communities, the functional profiles were significantly driven by the incorporation of chopped residues. Previous studies have demonstrated that chopped residues have a greater impact on soil functions in comparison to composted or pyrolyzed residues, affecting soil respiration (Huang et al., 2018), root exudates (Sun et al., 2020), and P cycling (Chen et al., 2017; Pu et al., 2023). The study confirmed the enhancement of C, N, P, and S cycling with chopped residue addition. Furthermore, composted residues were found to particularly promote C and S cycling. These observations can be attributed to the high carbon concentration in crop residues, where approximately 45% of the dry-weight biomass consists of carbon in forms such as lignin, cellulose, and hemicellulose that require decomposition by microorganisms (Blanco-Canqui and Lal, 2009; Ntonta et al., 2022). Consequently, genes associated with Glycoside Hydrolases, which aid in breaking down of plant cells and releasing fermentation products and CO_2_ (Vuong and Wilson, 2010), exhibited increased activity in decomposing organic matter. Genes related to the degradation of organonitrogen, organosulfur, and organophosphorus compounds were also upregulated. For example, the relative abundance of P cycling genes responsible for phosphate ester hydrolysis (such as *phoA*, *opd*, *ugpQ*, etc.) and energy capture and use (such as *ushA*, *pps*, *pcdK*, *etc*) increased under chopped or composted residue inputs. Alternatively, N and S cycling associated with amino acid metabolism (including *ureC*, *ureB*, *gln*, *nmo*, *etc.*) and organosulfur transformation (including *comC/D/E*, *dmsB/A*, *mddA*, *dddP*, *etc.*) increased following the addition of chopped or composted residues. Both genetic and functional diversity serve as indicators of soil health (Arias et al., 2005). Our results suggested that soil functional diversity exhibited greater sensitivity to different strategies of returning straws compared to genetic diversity.

### Decoupling of organic carbon and nutrient cycling with the addition of residues

Moreover, our findings indicated that the combination of chemical fertilizer and various residue treatments reduced the connectivity and density of modules between nutrient cycles in comparison to conventional fertilizers. Specifically, C, N, P, and S cycling exhibited significant correlations under conventional fertilization within four modules. The addition of chopped residues led to the formation of two modules related to the cycling of C, N and S. Composted residues resulted in two modules associated with C and S cycling, while pyrolyzed residues maintained a single module focusing on C, P, and S cycling. The accumulation of C during the decomposition of soil organic matter and crop residues is closely coupled with the enrichment of other nutrients like N, P, and K due to fertilization (Crowther et al., 2019; Mo et al., 2024). Studies have suggested that the soil organic C stock may become decoupled from the phosphorus stock with increased organic matter input or climate changes (Delgado-Baquerizo et al., 2013; Feng et al., 2019; Li et al., 2022). The coupling or decoupling of organic carbon and other nutrients has been attributed to microbial functions, although empirical evidence is limited.

The study outcomes offered insights into addressing the intricate coupling between nutrient cycles. Moreover, the research suggested that implementing different residue return strategies may cause diverse decoupling scenarios. On the one hand, residue introduction disrupted the inherent linkages of microbial-mediated C, N, and P cycling, potentially increasing leakages and losses from the system (Recous et al., 2019). The decoupled of soil nutrient cycles can negatively affect the provision of essential ecosystem services, including primary production and organic matter decomposition, both of which are defined as primary metrics of soil health (Delgado-Baquerizo et al., 2013; Pérez-Guzmán et al., 2021). This suggested the demand for a more innovative approach to achieve balanced fertilization (Jiang et al., 2021; Mo et al., 2024). On the other hand, S cycling maintained closely linked to C cycling across all residue treatments, highlighting its essential roles in photosynthetic processes, nitrogen utilization or protein biosynthesis (Chaudhary et al., 2023). This association presented promising opportunities for leveraging S cycling in future carbon sequestration approaches.

### All residue returning strategies led to ARGs enrichment while composted residues caused increased pathogens

The utilization of crop residues appeared to have a beneficial effect on reducing the presence of antibiotic residues and pathogens in soil, thereby enhancing soil health to some extent compared to the use of manure (Urra et al., 2019; Lu et al., 2021). However, our findings indicated that the application of residue amendments, particularly pyrolyzed residues, resulted in an enrichment of antibiotic resistance genes such as chloramphenicol, multidrug, pleuromutilin_tiamulin, sulfonamide, and puromycin. On one hand, the addition of residues provided nutrients for microbial growth, leading to an increase in the abundance of specific ARGs (Zhuang et al., 2021). On the other hand, residue additives may lead to the accumulation of ARGs in soil while decreasing their presence in plants (Shao et al., 2022), necessitating further investigation.

Furthermore, it is noteworthy that only composted residues led to an increase in the presence of pathogens such as *Colletotrichum*, *Cronobacter*, and *Yersinia*. Species from these genera have been identified as plant pathogens as well as potential human pathogens (Cornelis, 1998; Hyde et al., 2009; Forsythe, 2018), underscoring the importance of addressing this issue in future research.

Last but not least, it is important to noted that this study relied on metagenomic data obtained from a single sampling event. Future research should also focus on establishing the relationships between these findings and the actual phenomena through long-term continuous monitoring.

## Conclusion

Strong and significant correlations were identified among the significantly changed indices, leading to the formation of three distinct modules within the network. It is anticipated that modularity will rise with the specificity of links (Olesen et al., 2007). Acidobacteria exhibited a strong association with 50 genes related to C, N, P, and S cycling. Similarly, Thaumarchaeota, chloramphenicol, and the pathogen Colletotrichum displayed high connectivity with six genes involved in the cycling of C, N, P, and S. Therefore, these indices could serve as potential indicators of functional changes, in addition to key nodes such as *nrdB* and *ppd*, *asnB*, *metC*, and *GH102* in response to residue inputs.

**Figure 9:**
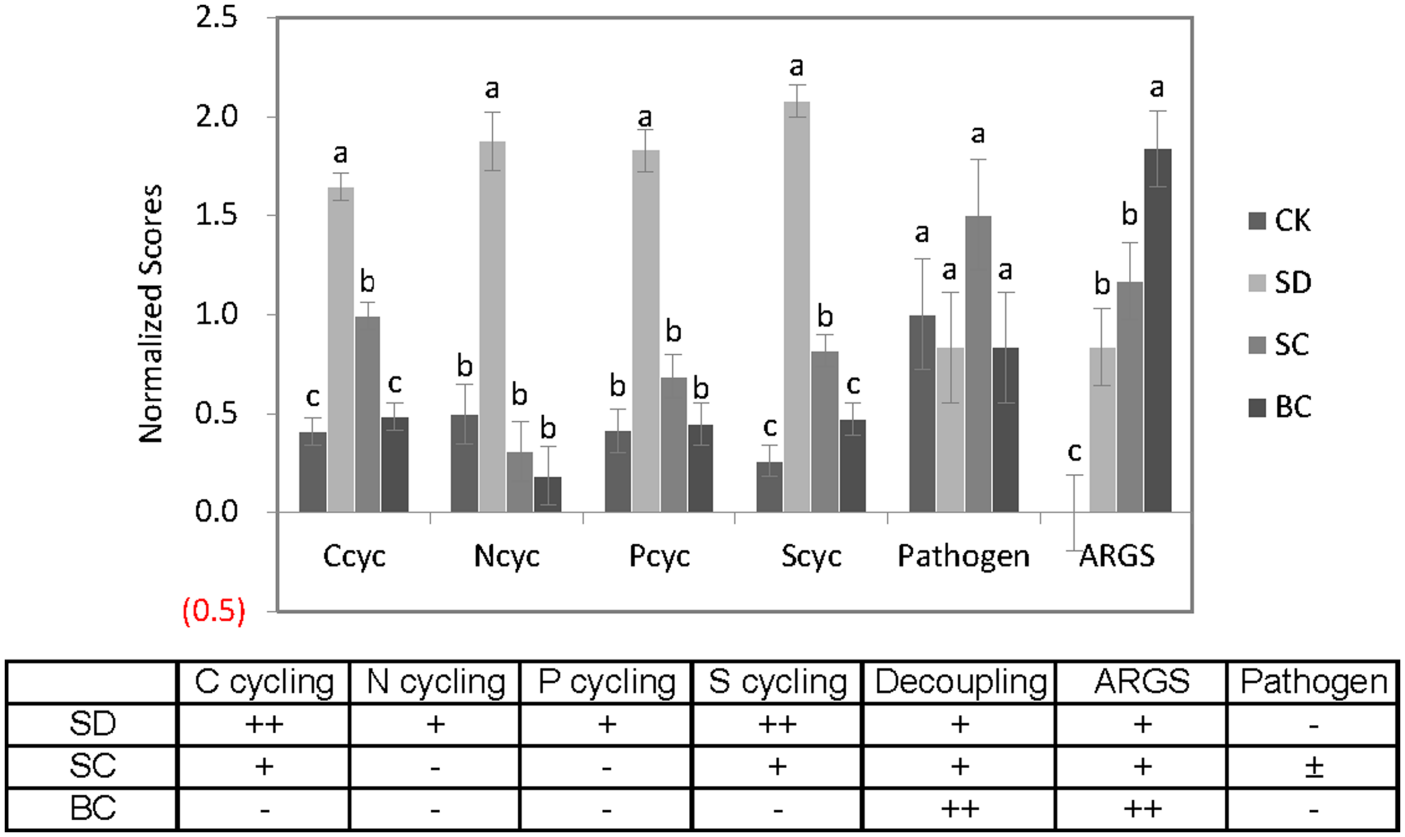
A comprehensive overview of the various indices studied across the treatments. CK, without addition of organic matter; BC, the addition of biochar converted from maize straw; SC, the addition of composted maize straw; SD, the addition of chopped maize straw. The lowercases indicate the significant difference between treatments one-way ANOVA with Duncan’s test (*p* < 0.05). the symbol “+” in the subsequent table signifies significant changes in each treatment.

In general, as illustrated in Figure 9, there was a notable increase in microbial capacity for C, N, P, and S cycling in response to chopped or composted residues, with a moderate level of decoupling between these processes. Both the newly added residues and the existing soil organic matter would undergo mineralization processes, which may surpass the necessary levels, potentially resulting in nutrient loss and depletion. Pyrolyzed residues exhibited minimal effect on microbial nutrient cycling, but displayed a high level of decoupling between these cycles. When considering ARGs and pathogens collectively, future research on soil health should prioritize the investigation of (1) variations in nutrient availability due to a heightened potential for mineralization, P cycling for optimal fertilization, and the presence of ARGs in soils under chopped residues; (2) N and P cycling for balanced fertilization, the presence of ARGs in soils, and pathogens in the context of composted residues; (3) N cycling for balanced fertilization and the presence of ARGs in soils in relation to pyrolyzed residues.

## Data Availability

The datasets presented in this study can be found in online repositories. The names of the repository/repositories and accession number(s) can be found in the article/Supplementary material.

## Footnotes

^1^ www.illumina.com

^2^ https://github.com/jstjohn/SeqPrep

^3^ https://github.com/najoshi/sickle

^4^ http://www.genome.jp/keeg/

^5^version 3.6.2, http://www.r-project.org
