# Supplementary material for: How do various strategies for returning residues change microbiota modulation: potential implications for soil health
Source: Front Microbiol. 2025 Jan 21;15:1495682. doi: 10.3389/fmicb.2024.1495682 (PMC11790580; doi:10.3389/fmicb.2024.1495682)
Supplement: Supplementary file 1 [file Data_Sheet_1.docx]

**How do various strategies for returning residues change microbiota modulation: potential implications for soil health**

Nan Jiang^1, 2^, Zhenhua Chen^1, 2^, Yi Ren^3^, Shichang Xie^4^, Zimeng Yao^1, 5^, Dongqi Jiang^1^, Yulan Zhang^1, 2, *^, Lijun Chen^1, 2, *^

^1^ CAS Key Laboratory of Forest Ecology and Silviculture, Institute of Applied Ecology, Chinese Academy of Sciences, Shenyang, Liaoning 110016, China;

^2^ Shenyang National Field Scientific Observation and Research Station of Farmland Ecosystem, Shenyang, Liaoning 110016, China;

^3^ Iotabiome Biotechnology Inc., Suzhou, Jiangxu 215000, China;

^4^ Suzhou Medical College, Soochow University, Suzhou 215123, China

^5^ University of Chinese Academy of Sciences, Beijing 100049, China.

*** Correspondence**

**Yulan Zhang**

Tel: (86)-24-8397-0357; Email: [ylzhang@iae.ac.cn](mailto:ylzhang@iae.ac.cn)

**Lijun Chen**

Tel: (86)-24-8397-0355; Email: [ljchen@iae.ac.cn](mailto:ljchen@iae.ac.cn)


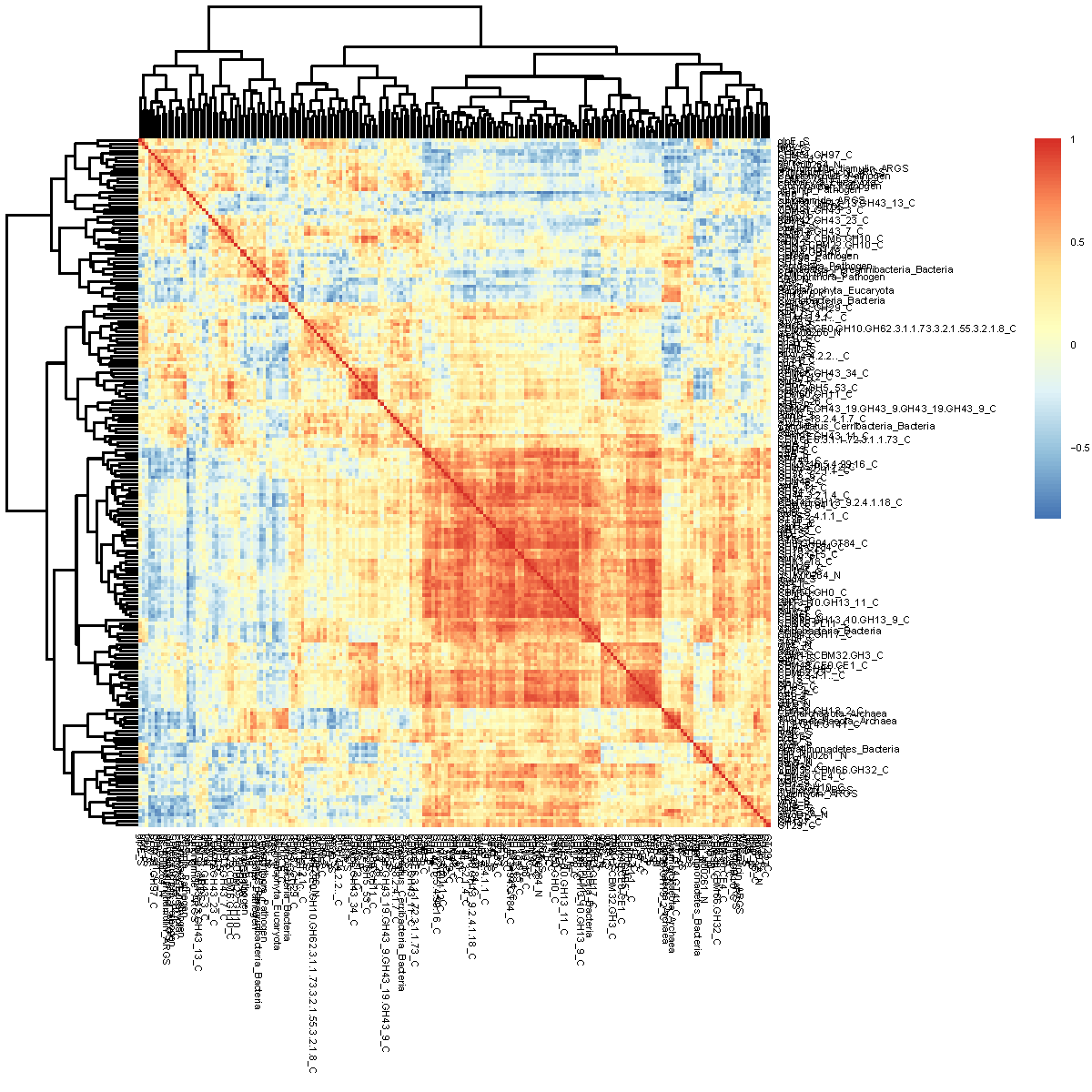


Figure S1 The correlations between the indices with significant changes in response to residue treatments.

Table S1 The chemical properties of different additives derived from straw

|  | pH (1:2.5 water) | Organic C (g kg^-1^) | Total N (g kg^-1^) | Total P (P_2_O_5_) (g kg^-1^) |
| --- | --- | --- | --- | --- |
| Straw | 6.75 | 0.42 | 4.15 | 3.71 |
| Biochar | 8.79 | 0.46 | 13.97 | 2.24 |
| Compost | 6.89 | 0.33 | 5.14 | 1.37 |

Table S2 The sequencing details for each replicate of every treatment

|  | Number of reads | Number of bases | contigs | ORFs^1^ |
| --- | --- | --- | --- | --- |
| CK1 | 101827165 | 15319503549 | 807823 | 1004974 |
| CK2 | 93357122 | 14043467489 | 676669 | 824157 |
| CK3 | 92856956 | 13964656362 | 627377 | 754124 |
| CK4 | 94984229 | 14288863056 | 643169 | 771989 |
| BC1 | 96918438 | 14580873205 | 598234 | 709141 |
| BC2 | 98240688 | 14782224091 | 827595 | 1022669 |
| BC3 | 96511902 | 14519773918 | 792090 | 983711 |
| BC4 | 95053818 | 14301364322 | 660050 | 800554 |
| SC1 | 96125046 | 14457770469 | 644842 | 790385 |
| SC2 | 94531496 | 14222870358 | 748508 | 913223 |
| SC3 | 105076101 | 15810556817 | 872963 | 1064883 |
| SC4 | 96532757 | 14523099248 | 691654 | 837632 |
| SD1 | 109267795 | 16438463955 | 752829 | 907816 |
| SD2 | 103037901 | 15499230740 | 676321 | 811006 |
| SD3 | 101212678 | 15228312732 | 868417 | 1074270 |
| SD4 | 108055074 | 16260166481 | 882061 | 1082609 |

^1^ ORFs: Open reading frames. CK: normal NPK fertilizer; BC: NPK plus biochar converted from straw; SC: NPK plus decomposed straw; SD: NPK plus straw.

| Table S3 The characteristics of networks in different treatments. | | | | |
| --- | --- | --- | --- | --- |
|  | CK | BC | SC | SD |
| clustering coefficient | 0.94 | 0.78 | 0.79 | 0.65 |
| network centralization | 0.06 | 0.12 | 0.12 | 0.11 |
| shortest paths | 200(20%) | 84(11%) | 110(13%) | 122(10%) |
| Avg. number of neighbors | 6.25 | 3.11 | 3.79 | 3.59 |
| Number of nodes | 32 | 27 | 29 | 34 |
| Network density | 0.20 | 0.12 | 0.14 | 0.11 |
| Network heterogeneity | 0.26 | 0.60 | 0.63 | 0.66 |

CK: normal NPK fertilizer; BC: NPK plus biochar converted from straw; SC: NPK plus decomposed straw; SD: NPK plus straw.
